# Rural Raccoons (*Procyon lotor*) Not Likely to Be a Major Driver of Antimicrobial Resistant Human *Salmonella* Cases in Southern Ontario, Canada: A One Health Epidemiological Assessment Using Whole-Genome Sequence Data

**DOI:** 10.3389/fvets.2022.840416

**Published:** 2022-02-25

**Authors:** Nadine A. Vogt, Benjamin M. Hetman, Adam A. Vogt, David L. Pearl, Richard J. Reid-Smith, E. Jane Parmley, Stefanie Kadykalo, Nicol Janecko, Amrita Bharat, Michael R. Mulvey, Kim Ziebell, James Robertson, John Nash, Vanessa Allen, Anna Majury, Nicole Ricker, Kristin J. Bondo, Samantha E. Allen, Claire M. Jardine

**Affiliations:** ^1^Department of Population Medicine, Ontario Veterinary College, Guelph, ON, Canada; ^2^Independent Researcher, Mississauga, ON, Canada; ^3^Centre for Foodborne, Environmental and Zoonotic Infectious Diseases, Public Health Agency of Canada, Guelph, ON, Canada; ^4^Quadram Institute Bioscience, Norwich, United Kingdom; ^5^National Microbiology Laboratory, Public Health Agency of Canada, Winnipeg, MB, Canada; ^6^Department of Medical Microbiology and Infectious Diseases, University of Manitoba, Winnipeg, MB, Canada; ^7^National Microbiology Laboratory, Public Health Agency of Canada, Guelph, ON, Canada; ^8^Public Health Ontario, Toronto, ON, Canada; ^9^Public Health Ontario, Kingston, ON, Canada; ^10^Department of Biomedical and Molecular Science, Queen's University, Kingston, ON, Canada; ^11^Department of Pathobiology, Ontario Veterinary College, Guelph, ON, Canada; ^12^Wyoming Game and Fish Department, Laramie, WY, United States; ^13^Department of Veterinary Sciences, University of Wyoming, Laramie, WY, United States; ^14^Canadian Wildlife Health Cooperative, Ontario Veterinary College, Guelph, ON, Canada

**Keywords:** antimicrobial resistance, foodborne illness, *Procyon lotor*, raccoon, *Salmonella*, whole-genome sequencing, wildlife

## Abstract

Non-typhoidal *Salmonella* infections represent a substantial burden of illness in humans, and the increasing prevalence of antimicrobial resistance among these infections is a growing concern. Using a combination of *Salmonella* isolate short-read whole-genome sequence data from select human cases, raccoons, livestock and environmental sources, and an epidemiological framework, our objective was to determine if there was evidence for potential transmission of *Salmonella* and associated antimicrobial resistance determinants between these different sources in the Grand River watershed in Ontario, Canada. Logistic regression models were used to assess the potential associations between source type and the presence of select resistance genes and plasmid incompatibility types. A total of 608 isolates were obtained from the following sources: humans (*n* = 58), raccoons (*n* = 92), livestock (*n* = 329), and environmental samples (*n* = 129). Resistance genes of public health importance, including *bla*_CMY−2_, were identified in humans, livestock, and environmental sources, but not in raccoons. Most resistance genes analyzed were significantly more likely to be identified in livestock and/or human isolates than in raccoon isolates. Based on a 3,002-loci core genome multi-locus sequence typing (cgMLST) scheme, human *Salmonella* isolates were often more similar to isolates from livestock and environmental sources, than with those from raccoons. Rare instances of serovars *S*. Heidelberg and *S*. Enteritidis in raccoons likely represent incidental infections and highlight possible acquisition and dissemination of predominantly poultry-associated *Salmonella* by raccoons within these ecosystems. Raccoon-predominant serovars were either not identified among human isolates (*S*. Agona, *S*. Thompson) or differed by more than 350 cgMLST loci (*S*. Newport). Collectively, our findings suggest that the rural population of raccoons on swine farms in the Grand River watershed are unlikely to be major contributors to antimicrobial resistant human *Salmonella* cases in this region.

## Introduction

Non-typhoidal *Salmonella* infections represent a major threat to public health worldwide (1). It has been estimated that non-typhoidal *Salmonella* cause roughly 93.8 million infections every year, resulting in approximately 155,000 deaths worldwide (2). The majority of infections result in self-limiting gastroenteritis, but invasive bloodstream infections do occur, with children, the elderly, and immunocompromised individuals at the greatest risk of developing life-threatening illness (3). The medical treatment of severe *Salmonella* infections can also become complicated by the presence of antimicrobial resistance [AMR; (4)]; resistant infections contribute to an increased risk of complications and death, in addition to prolonged recovery times (5, 6). The widespread use of antimicrobials in agriculture and in humans is thought to drive the selection of resistance and virulence among pathogenic organisms such as *Salmonella* (7, 8). Although wildlife species are increasingly being examined for their potential role in zoonotic diseases (9), their role in the epidemiology of *Salmonella* and AMR is not entirely clear. To achieve a greater understanding of the drivers of zoonotic infections in humans, and the transmission of AMR within the ecosystem, research approaches using multiple sampling sources, including wildlife and environmental samples, are increasingly being employed (10–14).

Recent work using comparative genomics has provided some epidemiological evidence that wild birds may contribute to the transmission of *Salmonella* between different human, animal, and environmental sources (15–18). With the vast majority of zoonotic disease investigations of *Salmonella* in wildlife focused on wild avian species, there is comparatively little work examining terrestrial wild mammals such as raccoons (*Procyon lotor*). A number of cross-sectional surveys have determined that raccoons can be asymptomatic carriers of non-typhoidal *Salmonella*, with serovars that overlap with those commonly responsible for causing illness in humans (19–22). Two studies which included genotypic assessments of *Salmonella* demonstrated the presence of identical pulsed-field gel electrophoresis (PFGE) patterns for certain human and raccoon isolates (23, 24).

In our previous work using whole-genome sequence (WGS) data to assess the potential for transmission of *Salmonella, E. coli* and AMR determinants between raccoons, soil, and manure pits on swine farms in southern Ontario, the identification of similar or identical subtypes (based on core-genome multi-locus sequence typing) among isolates from these different sources and farms was consistent with potential on-farm and between-farm transmission (25). Our present work assesses this same subset of samples originating from raccoons, soil, and manure pits on swine farms within the context of a greater geographic region in southern Ontario, by comparing those farm isolates to other *Salmonella* isolates from humans, livestock, and water sources collected during the same time period as part of public health surveillance programs. Our broad research aim was to address current knowledge gaps in our understanding of the role of raccoons in the epidemiology of human *Salmonella* infections in this region, as well as the movement of associated AMR determinants in this ecosystem, by using a combination of epidemiological modeling and WGS data analysis approaches. More specifically, our objective was to characterize *Salmonella* isolates from raccoons, humans, livestock and environmental sources, and to assess for patterns in the distribution of AMR determinants (i.e., genes, predicted plasmids) in these different sources using statistical modeling.

## Methods

### Dataset

Isolates examined within this study were obtained from three different collections of *Salmonella* cultures that were previously sampled through (1) a wildlife research study (21) and public health surveillance programs led by (2) the Public Health Agency of Canada, FoodNet Canada (https://www.canada.ca/en/public-health/services/surveillance/foodnet-canada.html), and (3) Public Health Ontario. *Salmonella* isolates collected through public health surveillance of human, livestock, and environmental sources were identified and included if the samples originated from the same geographic region and time period as the previous wildlife study (Wellington-Dufferin and Region of Waterloo Counties in southern Ontario, 2011–2013). The study region, which includes the cities of Waterloo, Kitchener, Guelph, Cambridge and surrounding areas, has a population of ~1 million people and is situated within a region of intensive agriculture. It is also located within the Grand River watershed (6,800 km^2^), the largest watershed in southern Ontario (21).

### Raccoon, Swine Manure Pit, and Soil Isolates

*Salmonella* isolates for this study component were obtained from a previous three-year repeated cross-sectional study of raccoons on swine farms and in conservation areas (21); only the subset of *Salmonella* isolates collected on swine farms were selected for sequencing. These samples included fecal swabs from live-trapped raccoons, soil samples collected adjacent to trapped raccoons, and swine manure pit samples. The sampling sites and methods used for live-trapping and processing of raccoons have been described previously (21).

### Livestock and Water Isolates

The recruitment and selection of farms for FoodNet Canada has previously been described (26). Livestock samples included pooled samples from swine, chickens (broilers and layers), and cattle (dairy and beef). Samples included both fresh fecal samples and manure pit samples for all species except for layers, from which only fresh fecal samples were obtained. Additional details about the sampling approach used are available in Flockhart et al. (26).

River water samples were obtained from five core water sites upstream of regional drinking water intake in the Grand River watershed. Beach water samples were obtained from local swimming sites in three different recreational areas in southern Ontario (Shade's Mills, Laurel Creek, Elora Gorge). Details regarding the approach and methods used to sample water from rivers and beaches have previously been described by Kadykalo et al. (27).

### Human Isolates

*Salmonella* isolates from human clinical cases collected by Public Health Ontario as part of the reportable disease surveillance program were cultured from blood, stool, and urine specimens (https://www.publichealthontario.ca/en/diseases-and-conditions/infectious-diseases/enteric-foodborne-diseases/salmonellosis).

### Previous Culture and Phenotypic Susceptibility Testing

Isolation of *Salmonella* was previously performed according to methods described in 'National Integrated Enteric Disease Surveillance Program: Sample Collection, Preparation and Laboratory Methodologies, January 2010' (https://www.phac-aspc.gc.ca/foodnetcanada/pdf/lab_sop-eng.pdf). One *Salmonella* isolate from each sample was sub-cultured and tested further. Presumptive-positive colonies were confirmed *via* biochemical reactions on triple sugar iron slant and urea slant agars (Becton, Dickinson). Additional confirmation was performed using *Salmonella* O Poly A-I & Vi antiserum (Becton, Dickinson). All isolates were previously tested for susceptibility to the following 15 antimicrobials by broth microdilution (Sensititre, CMV3AGNF panel, NARMS; Thermo Scientific): gentamicin (GEN), kanamycin (KAN), streptomycin (STR), amoxicillin-clavulanic acid (AMC), cefoxitin (FOX), ceftiofur (TIO), ceftriaxone (CRO), ampicillin (AMP), chloramphenicol (CHL), sulfisoxazole (SOX), trimethoprim-sulfamethoxazole (SXT), tetracycline (TCY), nalidixic acid (NAL), ciprofloxacin (CIP), and azithromycin (AZM). Susceptibility testing of livestock, wildlife, and environmental isolates was previously performed by the Antimicrobial Resistance Reference Laboratory, at the National Microbiology Laboratory (NML) at Guelph (Public Health Agency of Canada, Guelph, ON, Canada) in accordance with methods outlined by The Canadian Integrated Program for Antimicrobial Resistance Surveillance [CIPARS; (28)]. Isolates with intermediate resistance were classified as susceptible.

### Selection of Isolates for Whole-Genome Sequencing

All raccoon fecal, soil, and manure pit samples originating from swine farms in the previous wildlife study (21) were selected for sequencing. Previously sequenced human *Salmonella* isolates originating from the Wellington-Dufferin-Guelph or Region of Waterloo Public Health Counties in Ontario between 2011 and 2013 were included in the present study. Previous selection of human isolates for sequencing as part of CIPARS surveillance included routine surveillance of 11 serovars (4, [5], 12,i:-, Dublin, Enteritidis, Heidelberg, Infantis, Kentucky, Newport, Paratyphi A, Paratyphi B, Typhi, and Typhimurium), in addition to supplementary sequencing by request or for specific research projects investigating AMR [e.g., ciprofloxacin resistance, extended-spectrum beta-lactamases, *bla*_CMY−2_ in *S*. Heidelberg; (29–31)]. For further details regarding CIPARS methods, please see the Design and Methods section of the annual report (32). Previously sequenced *Salmonella* from water and livestock collected by FoodNet Canada were included in the present study if they had been collected within the Region of Waterloo sentinel site between 2011 and 2013. Previous selection for sequencing of livestock isolates collected by FoodNet Canada was done on the basis of metadata; up to five isolates with identical metadata were sequenced (i.e., same submitter, collection date, commodity, serotype, and phage-type, if applicable); contaminated isolates and isolates without a serotype were not eligible for sequencing.

### DNA Extraction, Whole-Genome Sequencing and Genome Assembly

Genomic DNA extractions were performed at the University of Guelph, or at the NML in Winnipeg, Manitoba. Briefly, *Salmonella* cultures were grown on Mueller Hinton agar and incubated at 35°C overnight. Cultures were then sent to the NML in Winnipeg for DNA extraction and sequencing, or these steps were performed on site, at the University of Guelph and the NML at Guelph, Ontario, respectively. Cultures of 1 ml *Salmonella* were used as input to the Qiagen DNEasy plant and tissue 96 kit, following manufacturer protocols (Qiagen, Hilden, Germany). Sequencing was performed using the Nextera XT libraries and Illumina MiSeq version 3 (600-cycle kit) or NextSeq550 platforms, according to the manufacturer's protocols. Raw reads were assembled using SPAdes (33), as part of the Shovill pipeline (version 1.0.1; https://github.com/tseeman/shovill) with the following settings: “–minlen 200 –mincov 2; –assembler spades; –trim”.

### Analysis of Whole-Genome Assemblies

Prediction of legacy multi-locus sequence types was performed using MLST (version 2.19.0; https://github.com/tseemann/mlst), which uses the Achtman 7-loci scheme for *Salmonella enterica* (https://pubmlst.org/mlst/). Core genome multi-locus sequence typing (cgMLST) of all isolates was performed using the “fairly simple allele calling” tool *fsac* (version 1.2.0; https://github.com/dorbarker/fsac) with the 3,002-loci Enterobase scheme (https://enterobase.warwick.ac.uk/). Isolates with 20 or more missing loci were considered poor quality and excluded from any further analyses. Isolates with greater than two missing genotypes for resistant phenotypic test results (by antimicrobial class), and a sequencing depth of <30 using Mash (34) were also excluded from further analyses. A threshold of *n* = 10 was used as the maximum number of allelic differences present for *Salmonella* to be considered as possibly belonging to the same strain, consistent with the threshold used by PulseNet (35). Population structure was visualized using cgMLST data and minimum spanning trees generated by the standalone GrapeTree software package [version 1.5; (36)], using the “MSTreeV2” algorithm, which accounts for missing data. For the minimum spanning tree visualizing the overall population of *Salmonella*, a lenient clustering threshold was used (*k* = 30) to provide a qualitative assessment of overlap between isolates from different sources, while minimizing unnecessary noise. An additional minimum spanning tree was constructed to examine isolate overlap for only serovars present in both human and raccoon isolates; a stricter clustering threshold (*k* = 10) was used to provide additional resolution and to minimize clustering of isolates from potentially different strains, consistent with the PulseNet threshold (35). Allelic differences between cgMLST profiles from different sources were calculated using R (version 3.6.3). R code is available at https://github.com/nadinevogt21/salmonella-human-raccoon-WGS-project. *In silico* identification of serovars was performed using the SISTR command-line tool (version 1.1.1; https://github.com/phac-nml/sistr_cmd), and default settings with the “centroid” allele database.

Acquired resistance genes were identified using CARD-RGI (database version 3.0.8; https://github.com/arpcard/rgi) which uses a curated database that includes optimized identity and coverage settings for each gene (37). The following setting was used: “–exclude nudge” (i.e., loose hits were not nudged to strict). Only acquired resistance genes identified with perfect and strict hits under the “protein homolog model” were reported; regulatory genes, genes requiring mutation for expression, and genes representing chromosomally encoded resistance genes were not reported. *In silico* identification of plasmid incompatibility groups was performed using Abricate (version 1.0.1; https://github.com/tseemann/abricate) and the PlasmidFinder database (updated May-17 2020) which uses the database from the Center for Genomic Epidemiology, Technical University of Denmark, DTU (https://cge.cbs.dtu.dk/services/PlasmidFinder/). Settings included nucleotide coverage of 70% and percent identity of 98%.

For raccoon and human isolates with fewer than 10 allelic differences based on the cgMLST scheme, Snippy (version 4.4.0; https://github.com/tseemann/snippy) was used to further distinguish genetic differences based on single-nucleotide polymorphisms (SNPs) using the entire genome, with default settings. *Salmonella enterica* subsp. *enterica* serovar Heidelberg str. SL476 (Accession No.: NC_011083.1) was used as a reference genome.

### Statistical Analyses

Multi-level logistic regression was used to model the odds of the presence of select plasmid incompatibility groups and acquired resistance genes found in *Salmonella* from different animal, human, and environmental sources. All statistical tests were performed using STATA (STATA Intercooled 14.2; StataCorp, College Station, Texas, USA). Only incompatibility groups or resistance genes with an overall prevalence of at least 10% and at most 90% were analyzed. Incompatibility types that were present only or predominantly (>95%) in one host-restricted serovar were reported descriptively only, due to the potential for complete confounding by serovar. Human and raccoon sources were retained as categories for analyses, and additional sources were grouped together into the following categories: livestock (cattle, swine, chicken), environmental (water, soil). Logistic regression models with source type as an independent variable were initially constructed using the “melogit” command, with a random intercept to account for clustering of isolates obtained from the same raccoon or swine manure pit (applicable to isolates from the wildlife research study). Site-level data for isolates obtained through FoodNet Canada were unavailable, thus, isolates that may have originated from the same farm or water source were treated as independent observations. For multi-level models that did not converge using “melogit”, the model was fitted using QR decomposition of parameters, using the command “meqrlogit”. All models were adjusted for the potential confounding effect of sampling year, if deemed appropriate. Sampling year was retained in the model based on the following criteria: it was statistically significant (*p* ≤ 0.05), or its removal resulted in a >20% change in the coefficient of the source type variable (38). To examine the fit of multi-level models, Pearson's residuals were assessed for outliers, and the best linear unbiased predictions (BLUPs) were assessed for normality and homoscedasticity. The random intercept was removed if the variance components were very small (<1 ×10^−3^) and if model fit was not improved based on changes to the Bayesian Information Criterion (BIC) value (38). If the random intercept was not retained in the final model, the model was fit using ordinary logistic regression. For ordinary logistic regression models, model fit was assessed using the Pearson's goodness-of-fit test, in addition to visual assessment of leverage, delta-beta, Pearson, and deviance residuals to identify any potential outliers. Outliers were investigated for recording errors, but otherwise left in the model. Exact logistic regression was used if low effective sample sizes posed estimation issues or to obtain median unbiased estimates if certain categories had zero positive observations; the score method was used to calculate *p*-values for these models. All tests were two-tailed, and a significance level of α = 0.05 was used.

## Results

### Description of Dataset

A total of 628 isolates were identified using the study criteria. After excluding isolates with 20 or more missing loci based on the 3,002-loci cgMLST scheme, 608 isolates were available for subsequent analyses from the following sources: human (*n* = 58), raccoon (*n* = 92), chicken (*n* = 214), cattle (*n* = 60), swine (from FoodNet Canada, *n* = 34), swine manure pit (from previous wildlife study; *n* = 21), soil (*n* = 45), water (*n* = 76), and beach (*n* = 8). Among isolates derived from cattle samples, these were roughly equally represented by beef (*n* = 29/60) and dairy animals (*n* = 31/60). The majority of chicken isolates were obtained from broilers (*n* = 204/214), with a handful of isolates from laying hens (*n* = 10/214). The subset of isolates obtained as part of the previous wildlife study (i.e., raccoon, soil, and swine manure pit samples) totaled 158; accession numbers for these isolates are available in Supplementary File 1. Isolates from all sources were evenly distributed across different sampling years: 2011 (36%), 2012 (34%), 2013 (30%). Since all human isolates that were included in this study exhibited phenotypic resistance to at least one antimicrobial (as part of the selection criteria for previous research projects), the prevalence of phenotypic multiclass resistance (to at least three drug classes) was highest in this source (65.5%, 95%CI: 51.8–77.5%). The prevalence of multiclass resistant and non-resistant isolates in the remaining source categories (all of which included phenotypically resistant and susceptible isolates, and were not selected based on phenotypic resistance) was as follows: livestock (10.6% multiclass resistant, 95%CI: 7.5–14.5%, 57.4% non-resistant, 95%CI: 51.9–62.8%), raccoon (2.2% multiclass resistant, 95%CI: 0.2–7.6%, 96.7% non-resistant, 95%CI: 90.8–99.3%), and environmental (3.1% multiclass resistant, 95%CI: 0.9–7.7%, 85.3% non-resistant, 95%CI: 77.9–90.9%). Results from previous phenotypic susceptibility testing of the 158 isolates from the swine farm subset is available in Bondo et al. (21).

### Serovar Distribution

**Figure 1:**
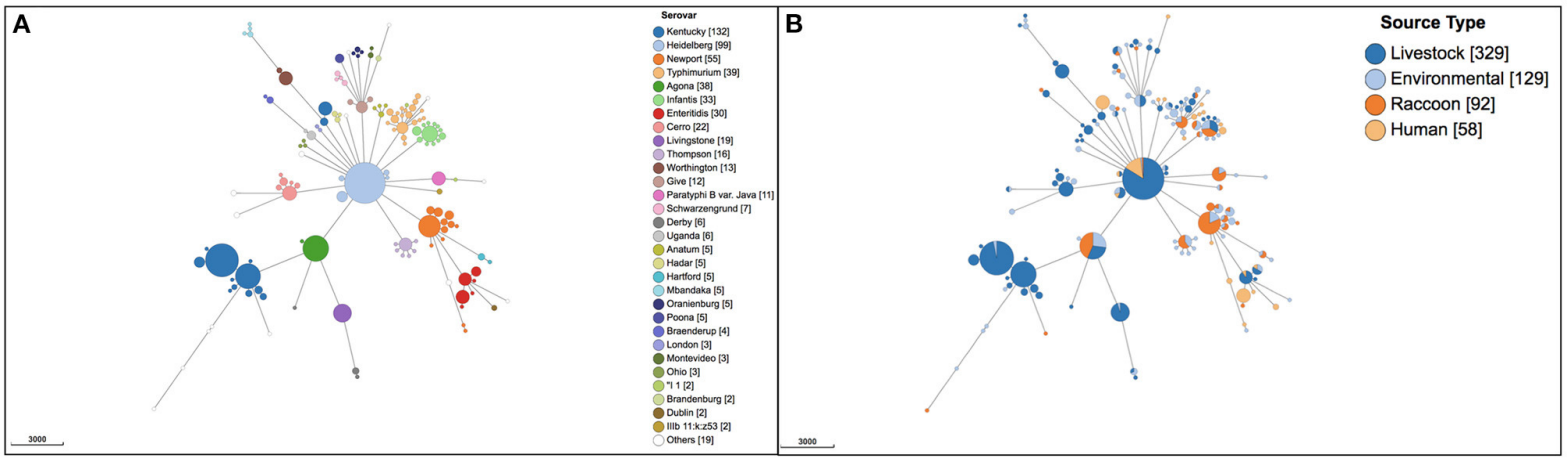
Population structure of 608 *Salmonella enterica* isolates from raccoons, livestock, humans, and environmental sources in southern Ontario, Canada based on 3002-loci cgMLST scheme from Enterobase. Minimum spanning tree created using *k* = *30* clustering threshold in *GrapeTree*. **(A)** Distribution of 45 serovars determined using SISTR. **(B)** Distribution of source types. Frequency counts are in square brackets. Bubble size is proportional to the number of isolates in each cluster, and each cluster contains isolates differing at a maximum of 30 cgMLST loci.

**Figure 2:**
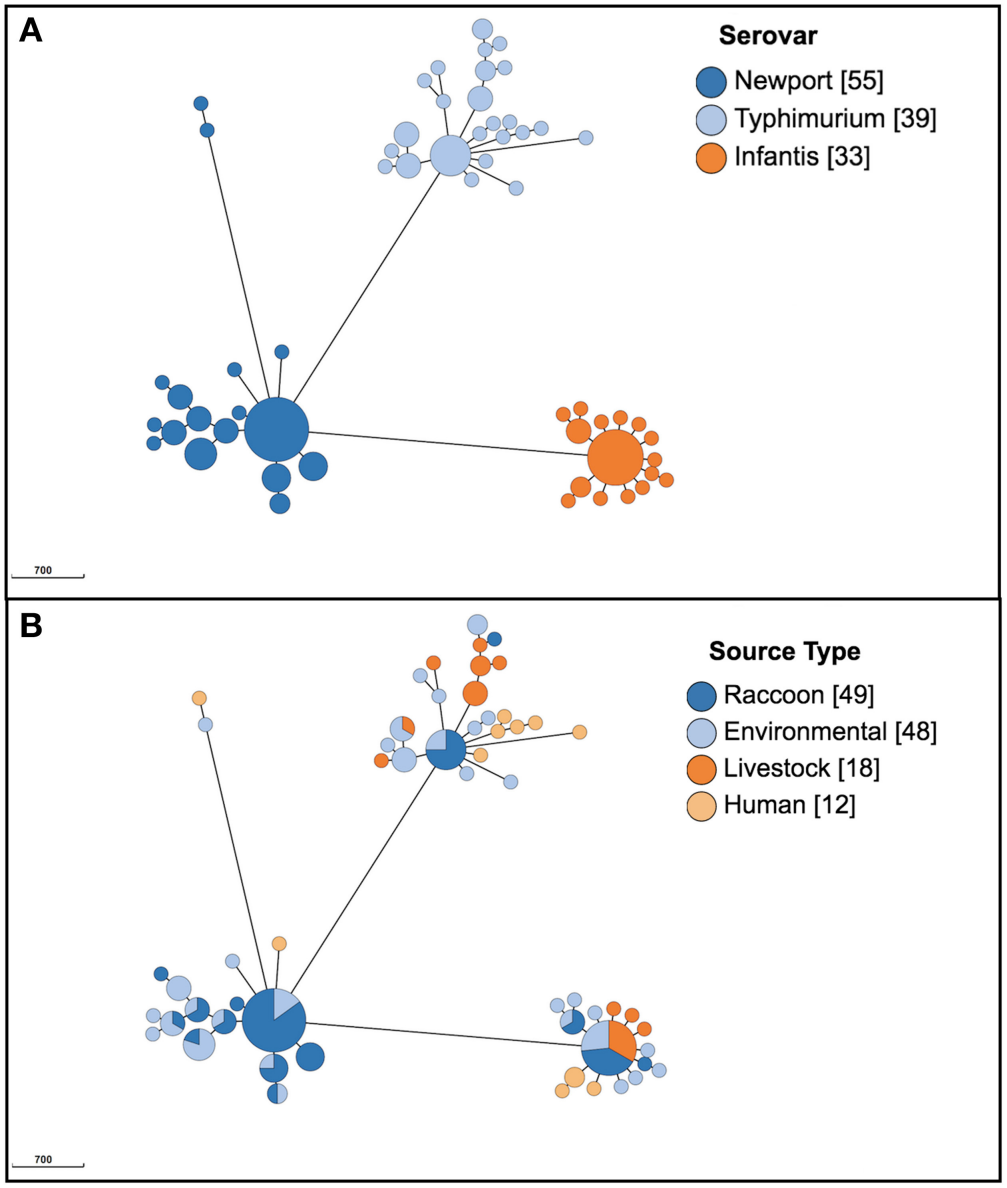
Population structure of 127 *Salmonella enterica* isolates from raccoons, livestock, humans, and environmental sources in southern Ontario, Canada based on 3002-loci cgMLST scheme from Enterobase, for serovars *S*. Newport, *S*. Typhimurium, and *S*. Infantis (only serovars identified both in human and raccoon isolates; serovars *S*. Heidelberg and *S*. Enteritidis are not depicted here since only one raccoon isolate was identified for each, and these are reported in text). Minimum spanning tree created using *k* = *10* clustering threshold in *GrapeTree*. **(A)** Population structure with serovars determined using SISTR. **(B)** Distribution of source types. Frequency counts are in square brackets. Bubble size is proportional to the number of isolates in each cluster, and each cluster contains isolates differing at a maximum of 10 cgMLST loci.

A total of 45 different serovars were identified, and the most commonly identified serovars among all isolates were *S*. Kentucky (*n* = 132), *S*. Heidelberg (*n* = 99), *S*. Newport (*n* = 55), *S*. Typhimurium (*n* = 39), *S*. Agona (*n* = 38), *S*. Infantis (*n* = 33) and *S*. Enteritidis (*n* = 30; Figure 1A). Population structure based on cgMLST and the distribution of source types for all isolates is illustrated in Figure 1B. For population structure of all isolates by detailed sampling source, see Supplementary Figure 1. Among raccoon isolates, the most frequently identified serovars were *S*. Newport (*n* = 33/92, 36%), *S*. Agona (*n* = 16/92, 17%), *S*. Infantis (*n* = 9/92, 9.8%), and *S*. Paratyphi var. Java (*n* = 9/92, 9.8%). The most prevalent serovars in human isolates were *S*. Enteritidis (*n* = 14/58, 24%), *S*. Heidelberg (*n* = 14/58, 24%), and *S*. Kentucky (*n* = 11/58, 19%); the latter two serovars were infrequently represented, or not present, among environmental and raccoon isolates, but were commonly identified in samples from chickens and cattle (Figure 1B). A heatmap of the distribution of serovars identified in humans in this study that were also identified in other sources is provided in Supplementary Table 1. *Salmonella* Enteritidis was isolated from all sources except for swine isolates. Several serovars that were infrequently identified in human isolates (<5%) were not identified in any other sample in our study (i.e., *S*. Litchfield, *S*. Dublin, *S*. Chester; Supplementary Table 1). Serovars present in both humans and raccoons, in decreasing frequency relative to raccoons, included: *S*. Newport, *S*. Infantis, *S*. Typhimurium, *S*. Heidelberg, and *S*. Enteritidis. The genetic relationships among isolates of *S*. Newport, *S*. Infantis, and *S*. Typhimurium are provided in Figure 2; since only one isolate each of *S*. Heidelberg and *S*. Enteritidis was identified in raccoons, these serovars were reported descriptively only. For detailed information regarding the distribution of all serovars by source, see Table 1.

**Table 1:** Distribution of *Salmonella enterica* serovars^a^ from raccoons, humans, livestock, and environmental sources in southern Ontario, Canada 2011–2013 (*n* = 608).

**Serovar**	**Source type**						**Total^**c**^ (%)**
	**Raccoon (*n* = 92)**	**Human (*n* = 58)**	**Environ-mental^**b**^ (*n* = 129)**	**Cattle (*n* = 60)**	**Swine (*n* = 55)**	**Chicken (*n* = 214)**	
*S*. Kentucky	0	11	3	4	0	114	132 (21.7)
*S*. Heidelberg	1	14	4	8	0	72	99 (16.3)
*S*. Newport	33	2	20	0	0	0	55 (9.0)
*S*. Typhimurium	7	6	16	4	5	1	39 (6.4)
*S*. Agona	16	0	11	1	9	1	38 (6.2)
*S*. Infantis	9	4	12	1	6	1	33 (5.4)
*S*. Enteritidis	1	14	2	3	0	10	30 (4.9)
*S*. Cerro	0	0	3	19	0	0	22 (3.6)
*S*. Livingstone	0	0	1	0	10	8	19 (3.1)
*S*. Thompson	6	0	10	0	0	0	16 (2.6)
*S*. Worthington	0	0	0	1	12	0	13 (2.1)
*S*. Give	0	0	8	4	0	0	12 (2.0)
*S*. Paratyphi B var. Java	9	0	2	0	0	0	11 (1.8)
*S*. Schwarzengrund	1	0	4	0	2	0	7 (1.1)
*S*. Derby	0	0	2	1	3	0	6 (0.9)
*S*. Uganda	0	0	0	5	1	0	6 (0.9)
*S*. Poona	1	0	2	0	1	1	5 (0.8)
*S*. Anatum	0	2	1	0	0	2	5 (0.8)
*S*. Oranienburg	1	0	1	3	0	0	5 (0.8)
*S*. Mbandaka	0	0	3	0	2	0	5 (0.8)
*S*. Hartford	2	0	3	0	0	0	5 (0.8)
*S*. Hadar	1	0	3	1	0	0	5 (0.8)

a *Determined in silico using SISTR*.

b *Includes water isolates obtained through FoodNet Canada surveillance, as well as soil isolates obtained from a wildlife study (21)*.

c *Other serovars identified with fewer than five isolates were: S. Braenderup (n = 4), S. London (n = 3), S. Montevideo (n = 3), S. Ohio (n = 3), S. Senftenberg (n = 2), S. Kiambu (n = 2), S. Dublin (n = 2), S. Brandenburg (n = 2), S. Litchfield (n = 2), S. Orion (n = 2), S. IIIb 11:k:z53 (n = 2), S. Mikawasima (n = 2), S. I 1,4, [5], 12:i:- (n = 1), S. Tennessee (n = 1), S. Berta (n = 1), S. I 1,4, [5], 12:b:- (n = 1), S. Molade (n = 1), S. Saintpaul (n = 1), S. Rissen (n = 1), S. Stanley (n = 1), S. Holcomb (n = 1), S. Bovismorbificans (n = 1), S. Chester (n = 1)*.

### MLST Results

A total of 54 sequence types were identified, and the distribution of the 11 most common sequence types by source is provided in Table 2. Seven isolates were not typeable by MLST (two raccoon, one soil, two water, one swine, one chicken) due to a missing allele, or a partial match. The following internationally recognized sequence types (39) were identified in our study population: *S*. Heidelberg ST15 (*n* = 95), *S*. Typhimurium ST19 (*n* = 33), *S*. Infantis ST32 (*n* = 32), ST198 *S*. Kentucky (*n* = 15), ST96 *S*. Schwarzengrund (*n* = 7), ST10 *S*. Dublin (*n* = 2), ST45 *S*. Newport (*n* = 2), and ST65 *S*. Brandenburg (*n* = 2). As chickens were the most common source type in this study, sequence types from poultry-adapted serovars such as *S*. Kentucky ST152, and *S*. Heidelberg ST15 were most common (Table 2). *Salmonella* Kentucky isolates identified in humans (exclusively ST198) were infrequently identified in chicken isolates (*n* = 4; Table 2). Among *S*. Kentucky isolates from chickens, the vast majority (*n* = 109/114) of these were ST152, and the remaining isolates were ST198 (*n* = 4/114); one sample could not be typed due to a partial or missing allele. Most *S*. Newport isolates from raccoons and environmental sources were ST350, whereas the two human *S*. Newport isolates identified in our study were both ST45. Certain sequence types, including *S*. Infantis ST32 and *S*. Typhimurium ST19, were identified in every source. Other sequence types were identified only or predominantly in certain animal sources, but could also be found in water samples: *S*. Cerro ST367 and *S*. Give in cattle. For detailed information regarding the distribution of sequence types by source, see Supplementary Table 2.

**Table 2:** Counts of most frequently identified sequence types of *Salmonella enterica* isolates from raccoons, humans, livestock, and environmental sources in southern Ontario, Canada 2011–2013 (*n* = 608).

			**Counts of most common sequence types (all sources)**										
**Source type**		**Total no. of isolates collected (*n*)**	**ST152 (*S*. Kentucky)**	**ST15 (*S*. Heidelberg)**	**ST350 (*S*. Newport)**	**ST13 (*S*. Agona)**	**ST19 (*S*. Typhimurium)**	**ST32 (*S*. Infantis)**	**ST11 (*S*. Enteritidis)**	**ST367 (*S*. Cerro)**	**ST638 (*S*. Livingstone)**	**ST26 (*S*. Thompson)**	**ST198 (*S*. Kentucky)**
	Human	58	0	13	0	0	1	3	14	0	0	0	11
Wildlife	Raccoon	92	0	1	31	16	7	9	1	0	0	6	0
Livestock	Cattle	60	4	8	0	1	4	1	3	19	0	0	0
	Swine	55	0	0	0	9	5	6	0	0	10	0	0
	Chicken	214	109	69	0	1	1	1	10	0	8	0	4
Environmental	Soil	45	0	0	11	8	6	5	1	0	1	4	0
	Water	76	3	4	5	3	7	5	1	3	0	5	0
	Beach	8	0	0	1	0	2	2	0	0	0	1	0
Total (*n*)		608	116	95	48	38	33	32	30	22	19	16	15

*Distribution of all sequence types identified in this study is available in Supplementary Table 2*.

### Population Structure

Serovars identified among both human and raccoon isolates were: *S*. Enteritidis, *S*. Heidelberg, *S*. Infantis, *S*. Newport, and *S*. Typhimurium (Figure 2). Based on our previously specified cluster threshold of 10 loci, none of the human and raccoon isolates clustered together for any of the serovars present in both host species: *S*. Infantis, *S*. Newport, and *S*. Typhimurium isolates with a minimum of 73, 359 and 76 different loci, respectively (Figure 2). Single antimicrobial susceptible isolates of *S*. Enteritidis ST11 and *S*. Heidelberg ST15 were identified in raccoons and differed from human isolates by a minimum of 61 and four loci, respectively. Further analysis of the single raccoon *S*. Heidelberg ST15 isolate compared to the five closely related human isolates (<10 cgMLST loci different) revealed a minimum of 15 and a maximum of 21 SNPs that differed between these isolates.

*Salmonella* Infantis was identified in all source types; all 33 isolates were within 107 loci of one another, whereas all non-human sources containing *S*. Infantis could be grouped together at a lower threshold of 86 loci. The minimum number of allelic differences to the most closely related *S*. Infantis human isolate was similar for raccoons (73 loci) and livestock (72 loci) but was lower for environmental samples (56 loci). In contrast, the 30 *S*. Enteritidis isolates which were found in all source types (but predominantly humans and livestock) clustered together at higher threshold of 441 loci. Among *Salmonella* Enteritidis isolates (*n* = 30), livestock and environmental isolates displayed greater similarity to human isolates (a minimum of 12 and 16 allelic differences, respectively) compared to the single raccoon *S*. Enteritidis isolate (at least 61 allelic differences). *Salmonella* Typhimurium, also isolated from all sources (*n* = 39 isolates), displayed a high clustering threshold for all isolates, with all but two isolates grouped together at a threshold of 377 loci. The minimum numbers of allelic differences for *S*. Typhimurium isolated from raccoons, livestock, and environmental samples compared with human isolates were comparable (76, 79, and 69 loci, respectively). Serovars such as *S*. Heidelberg (*n* = 99) that were predominantly isolated from chickens displayed a considerably lower clustering threshold, together with isolates from humans, water, cattle, and the single raccoon *S*. Heidelberg isolate (*n* < 37 allelic differences for 85% of isolates). Identical *S*. Heidelberg were identified between livestock (chicken) and human samples (0 allelic differences), whereas a minimum of at least 16 and four allelic differences were noted for environmental isolates and the single raccoon isolate, respectively.

Two distantly related clusters of *S*. Kentucky were identified (>2,700 allelic differences). *Salmonella* Kentucky (*n* = 132) identified in environmental samples differed from the nearest human isolates by 2,758 loci, compared to the minimum of 139 loci that differed between livestock and human isolates (*S*. Kentucky was not identified among raccoon isolates). Finally, for serovars such as *S*. Newport (*n* = 55) that were mainly identified in environmental sources (e.g., soil) and raccoon isolates, many had identical cgMLST types (0 allelic differences). The two human *S*. Newport isolates differed from the most closely related raccoon *S*. Newport isolate at 359 and 2,592 loci, respectively. In contrast, the minimum number of allelic differences between *S*. Newport from environmental isolates and human isolates was lower (126 loci).

### *In silico* Determination of Plasmid Incompatibility Groups and Acquired AMR Genes

A total of 65 acquired resistance genes and 43 incompatibility groups were identified among all isolates. Commonly identified resistance genes with an overall prevalence >10% were: *fosA7, tet(B), aph(6)-Id, aph(3”)-Ib, aac(6')-Iaa*, and *bla*_CMY−2_ (Table 3). Human isolates contained the greatest diversity of resistance genes, with 58/65 genes identified. Other sources contained fewer resistance genes, in decreasing order: livestock (*n* = 29/65 genes), environmental (*n* = 27/65 genes), and raccoon isolates (*n* = 9/65 genes). A number of human and livestock isolates, along with three water isolates, contained *bla*_CMY−2_, which confers the cephamycinase-producing phenotype. Class A and C beta-lactamase genes responsible for conferring the extended-spectrum beta-lactamase (ESBL) phenotype (e.g., *bla*_CTX−M−14_, *bla*_CTX−M−65_) were only identified among human isolates. One human isolate was contained a *mcr-1* gene conferring resistance to colistin. Two human isolates with the ACSSuT (ampicillin, chloramphenicol, streptomycin, sulfamethoxazole, and tetracycline) resistance pattern were identified, one in a ciprofloxacin-resistant *S*. Typhimurium, and another in a *S*. Infantis resistant to third-generation cephalosporins.

**Table 3:** Frequencies of acquired antimicrobial resistance genes identified using whole-genome sequence data from *Salmonella enterica* isolates from raccoons, humans, livestock, and environmental sources in southern Ontario, Canada, 2011–2013 (*n* = 608).

**Antimicrobial group**	**Resistance gene**	**ARO^**†**^**	**Human (*n* = 58)**	**Livestock (*n* = 329)**	**Raccoon (*n* = 92)**	**Environment (*n* = 129)**	**Total (%)**
Aminoglycoside	aac(6')-Ib-cr^*^	3002547	16	0	0	0	16 (2.6%)
	*aac(6')-Ib8*	3002579	1	0	0	0	1 (0.2%)
	*aac(6')-Iaa*	3002571	17	35	8	22	82 (13.5%)
	*aac(3)-Id*	3002529	10	0	0	0	10 (1.6%)
	*aac(3)-IId*	3004623	3	1	0	1	5 (0.8%)
	*aac(3)-IV*	3002539	5	1	0	0	6 (1.0%)
	*aac(3)-VIa*	3002540	1	1	0	0	2 (0.3%)
	*aadA1*	3002601	2	0	0	0	2 (0.3%)
	*aadA2*	3002602	1	2	0	3	6 (1.0%)
	*aadA3*	3002603	2	0	0	0	2 (0.3%)
	*aadA4*	3002604	0	5	1	0	6 (1.0%)
	*aadA7*	3002607	10	0	0	0	10 (1.6%)
	*aadA16*	3002616	13	0	0	0	13 (2.1%)
	*ant(2”)-Ia*	3000230	2	1	0	0	3 (0.5%)
	*ant(3”)-IIa*	3004089	7	12	1	4	24 (3.9%)
	*aph(6)-Id*	3002660	9	72	1	6	88 (14.5%)
	*aph(3')-Ia*	3002641	4	6	0	1	11 (1.8%)
	*aph(3”)-Ib*	3002639	5	71	0	3	79 (13.0%)
	*aph(4)-Ia*	3002655	5	1	0	0	6 (1.0%)
Beta-lactam	*bla*_CMY−2_	3002013	17	57	0	3	77 (12.7%)
	*bla*_CTX−M−14_	3001877	2	0	0	0	2 (0.3%)
	*bla*_CTX−M−65_	3001926	3	0	0	0	3 (0.5%)
	*bla*_TEM−1_	3000873	14	15	0	4	33 (5.4%)
	*bla*_CARB−3_	3002242	0	5	0	2	7 (1.2%)
	*bla*_DHA−1_	3002132	1	0	0	0	1 (0.2%)
	*bla*_OXA−1_	3001396	3	0	0	0	3 (0.5%)
Lincosamide	*linG*	3002879	1	0	0	0	1 (0.2%)
	*lnuG*	3004085	0	3	0	0	3 (0.5%)
Macrolide	*mphA*	3000316	1	0	0	0	1 (0.2%)
Nucleoside	*SAT-2*	3002895	1	1	0	0	2 (0.3%)
Folate pathway inhibitors	*dfrI*	3004645	0	3	0	0	3 (0.5%)
	*dfrA1*	3002854	1	0	0	0	1 (0.2%)
	*drfA12*	3002858	1	0	0	1	2 (0.3%)
	*dfrA14*	3002859	5	0	0	0	5 (0.8%)
	*dfrA23*	3003019	1	0	0	0	1 (0.2%)
	*dfrA25*	3003020	0	0	0	2	2 (0.3%)
	*dfrA27*	3004550	13	0	0	0	13 (2.1%)
	*sul1*	3000410	31	11	2	4	48 (7.9%)
	*sul2*	3000412	10	2	0	1	13 (2.1%)
	*sul3*	3000413	2	3	0	1	6 (1.0%)
Phenicol	*floR*	3002705	13	7	0	3	23 (3.8%)
	*catB3*	3002676	3	0	0	0	3 (0.5%)
	*cmlA1*	3002693	2	0	0	1	3 (0.5%)
	*cmlA5*	3002695	1	0	0	1	2 (0.3%)
	*catII* from *Escherichia coli* K12	3004656	1	0	0	0	1 (0.2%)
Polymyxin	*mcr1.1*	3003689	1	0	0	0	1 (0.2%)
Quinolone	*qnrA1*	3002707	1	0	0	0	1 (0.2%)
	*qnrB4*	3002718	1	0	0	0	1 (0.2%)
	*qnrB6*	3002720	13	0	0	0	13 (2.1%)
	*qnrB19*	3002734	1	0	0	0	1 (0.2%)
	*qnrB20*	3002735	0	0	0	2	2 (0.3%)
	*qnrS1*	3002790	5	0	0	1	6 (1.0%)
	*qacH*	3003836	2	0	0	1	3 (0.5%)
	oqxA^**^	3003922	3	0	0	0	3 (0.5%)
	oqxB^**^	3003923	2	0	0	0	2 (0.3%)
	adeF^**^	3000777	1	0	0	0	1 (0.2%)
Rifamycin	*arr-3*	3002848	16	0	0	0	16 (2.6%)
Fosfomycin	*fosA3*	3002872	2	0	0	0	2 (0.3%)
	*fosA7*	3004113	14	94	18	17	143 (23.5%)
	*mdtG*	3001329	1	2	1	4	8 (1.3%)
Tetracycline	*tet(A)*	3000165	34	12	2	9	57 (9.4%)
	*tet(B)*	3000166	4	75	1	2	82 (13.5%)
	*tet(C)*	3000167	0	10	0	0	10 (1.6%)
	*tet(D)*	3000168	2	5	0	2	9 (1.5%)
	*tet(M)*	3000186	2	1	0	1	4 (0.6%)

† *Antibiotic resistance ontology accession number as listed in the comprehensive antibiotic resistance database*.

* *Gene may also confer fluoroquinolone resistance*.

** *Gene may also confer resistance to tetracyclines*.

Few resistance genes were identified among raccoon isolates (Table 3), corresponding with the *n* = 3/92 isolates that demonstrated phenotypic resistance (one isolate with STR-TCY, and two isolates with SOX-STR-TCY). Most of the resistance genes identified in raccoon isolates conferred resistance to aminoglycosides (*n* = 11/35 genes) and fosfomycin (*n* = 19/35 genes); no beta-lactam, phenicol, or quinolone resistance genes were identified in isolates from any of these animals (Table 3). In contrast, environmental isolates contained resistance genes to several major classes of antimicrobials (including beta-lactamases, phenicols, and quinolones; Table 3). The most commonly identified plasmid incompatibility groups are presented in Table 4. Some incompatibility groups were only identified in certain sources (e.g., ColE10 in swine), or appeared predominantly in certain sources (e.g., IncX1-3 in chicken isolates, IncFiip96a in raccoon and environmental isolates). Other incompatibility groups were identified in all source types (e.g., IncI1-Igamma, IncFIIS). For a summary of test sensitivity and specificity of *in silico* AMR prediction using phenotypic susceptibility testing results as the gold standard, see Supplementary Table 3. The overall sensitivity and specificity of *in silico* identification of AMR genes were 96.3 and 97.0%, respectively.

**Table 4:** Distribution of predicted plasmids identified using whole-genome sequence data by source type for *Salmonella enterica* isolates from raccoons, humans, livestock, and environmental sources in southern Ontario, Canada 2011–2013 (*n* = 608).

**Incompatibility type**	**Source type**						**Total^**b**^ (%)**
	**Raccoon (*n* = 92)**	**Human (*n* = 58)**	**Environmental^**a**^ (*n* = 129)**	**Cattle (*n* = 60)**	**Swine (*n* = 55)**	**Chicken (*n* = 214)**	
IncX1-1	22	28	11	9	0	80	150 (24.7%)
IncI1-Igamma	2	14	11	28	3	75	133 (21.9%)
IncFIIS	46	15	35	8	4	11	119 (19.6%)
IncX1-3	0	0	2	2	0	101	105 (17.3%)
ColRNAI	4	10	10	19	5	47	95 (15.6%)
ColpVC	1	6	13	22	2	36	80 (13.2%)
IncFiip96a	44	0	26	2	0	0	72 (11.8%)
IncFIB(AP001918)	0	1	2	1	1	64	69 (11.3%)
ColpHAD28	4	5	10	11	15	15	60 (9.9%)
IncFIB(S)	2	14	9	6	4	10	45 (7.4%)
IncX3	21	1	6	1	0	8	37 (6.1%)
ColYe4449	15	0	9	1	9	0	34 (5.6%)
Col156	1	1	3	15	1	3	24 (3.9%)
IncN1	0	14	2	0	0	1	17 (2.8%)
Col440II	2	4	4	1	1	3	15 (2.5%)
Col8282	0	4	3	1	0	4	12 (2.0%)
Col440I	2	0	2	0	2	5	11 (1.8%)
Pkpccav1321	1	4	0	0	0	5	10 (1.6%)
ColE10	0	0	0	0	10	0	10 (1.6%)

a *Includes beach and water isolates obtained through FoodNet Canada surveillance, as well as soil isolates obtained from a wildlife study (21)*.

b *Plasmid incompatibility types identified in fewer than 10 isolates included: IncH(1a) (n = 2), IncHI2 (n = 9), IncC (n = 7), IncI2(delta) (n = 6), IncFIB (pN55391) (n = 4), IncQ2 (n = 4), IncFII(pHN7a8) (n = 3), IncFII (n = 3), IncFII(p14) (n = 2), IncY1 (n = 3), Col(MP18) (n = 2), IncI1a (n = 2), IncHI1(bR27) (n = 2), IncFIA(HI1) (n = 2), IncFIB(pB171) (n = 2), IncX4 (n = 1), IncN2 (n = 1), Col(MG828) (n = 1), IncFICFII (n = 1), IncI(gamma) (n = 1), IncFIA (n = 1), pENTAS02 (n = 1), Incx4(2) (n = 1), IncFIB(pHCM2) (n = 1)*.

### Associations Between Source Type and Carriage of AMR Genes and Plasmid Incompatibility Groups

Except for IncI1-Igamma, all incompatibility groups and resistance genes examined had a significant association with source type. Certain incompatibility groups were significantly more likely to be identified in human or livestock than in raccoon or environmental isolates (i.e., IncX1-1, colRNAI, colpVC, IncI1-Igamma), whereas others were significantly more likely to be identified in raccoon and environmental isolates compared to human and livestock isolates (i.e., IncFIIS, IncFiip96a; Tables 5, 6). Incompatibility groups IncX1-3 and IncFIB(AP001918) were not modeled since they were exclusively, or almost exclusively (>95%), identified in one serovar (*Salmonella* Kentucky), and the vast majority (>85%) of these isolates originated from broiler chickens. Most resistance genes analyzed were significantly more likely to be identified in livestock and/or human isolates compared with environmental and/or raccoon isolates (i.e., *tetB*, $aac\left(6^{\prime }\right)-Iaa,aph\left(6\right)-Id,aph\left(3^{\prime \prime }\right)-Ib,bla_{\text{CMY}-2}$). In particular, the odds of identifying *tet(B)* and *aph(3”)-Ib* were highest in livestock isolates, and the odds of identifying *bla*_CMY−2_ were highest in human isolates (Tables 5, 6). The odds of identifying *bla*_CMY−2_ were also significantly greater in livestock isolates compared to raccoon or environmental isolates (Table 6). Random intercepts were not retained in several models since variance components were extremely small (i.e., ColpVC, *tetB, aac6'-Iaa, aph-6-Id*, fosA7) or exact logistic regression was used (i.e., *bla*_CMY−2_*, aph3”-Ib*, IncFiip96a).

**Table 5:** Logistic regression models assessing the association between source type and the occurrence of select antimicrobial resistance genes and predicted plasmids in *Salmonella enterica* isolates from raccoons, humans, livestock, and environmental sources in southern Ontario, Canada 2011–2013 (*n* = 608).

	**IncX1-1** ^**a**^		**IncI1-Igamma** ^**a**^		**IncFIIS** ^**a**^		**ColRNAI** ^**a**^		**ColpVC** ^**b**^ ^,^ ^**d**^	
**Source type**	**OR (95%CI)**	***p*-value**	**OR (95%CI)**	***p*-value**	**OR (95%CI)**	***p*-value**	**OR (95%CI)**	***p*-value**	**OR (95%CI)**	***p*-value**
Human	REF	**0.019 (global)**	REF	**0.092 (global)**	REF	**<0.001 (global)**	REF	**0.010 (global)**	REF	**<0.001 (global)**
Livestock	0.21 (0.06–0.79)	0.021	1.93 (0.63–5.90)	0.249	0.20 (0.08–0.50)	0.001	1.86 (0.44–7.80)	0.398	2.32 (0.94–5.75)	0.068
Raccoon	0.15 (0.03–0.76)	0.022	0.03 (0.00–0.60)	0.021	3.14 (1.22–8.09)	0.018	0.10 (0.01–0.78)	0.029	0.10 (0.01–0.85)	0.035
Environment	0.02 (0.00–0.24)	0.002	0.18 (0.03–0.98)	0.047	1.07 (0.50–2.30)	0.856	0.22 (0.04–1.23)	0.085	0.96 (0.34–2.70)	0.943
	**IncFiip96a** ^**c**^		***tet*(*B*)^b^^,^^d^**		***aac*(6′)−*Iaa*^d^**		***aph*(6)−*Id*^b^^,^^d^**		***aph*(3″)−*Ib*^c^**	
**Source type**	**OR (95%CI)**	***p*** **-value**	**OR (95%CI)**	***p*** **-value**	**OR (95%CI)**	***p*** **-value**	**OR (95%CI)**	***p*** **-value**	**OR (95%CI)**	***p*** **-value**
Human	REF	**<0.001 (global)**	REF	**<0.001 (global)**	REF	**0.001 (global)**	REF	**<0.001 (global)**	REF	**<0.001 (global)**
Livestock	0.42^*^ (0.03–∞)	0.999	4.23 (1.47–12.16)	0.007	0.29 (0.15–0.56)	<0.001	1.62 (0.75–3.47)	0.218	2.91 (1.11–9.68)	0.030
Raccoon	73.63^*^ (12.72–∞)	<0.001	0.12 (0.01–1.15)	0.067	0.23 (0.09–0.58)	0.002	0.05 (0.01–0.45)	0.007	0.09^*^ (0.00–0.66)	0.008
Environment	20.46^*^ (3.50–∞)	<0.001	0.20 (0.04–1.15)	0.072	0.49 (0.24–1.03)	0.059	0.26 (0.09–0.77)	0.015	0.25 (0.04–1.36)	0.062
	$bla_{\text{CMY}-2}^{c}$		***fosA*7^d^**							
**Source type**	**OR (95%CI)**	***p*** **-value**	**OR (95%CI)**	***p*** **-value**						
Human	REF	**<0.001 (global)**	REF	**0.003 (global)**						
Livestock	0.51 (0.26–1.02)	0.045	1.26 (0.66–2.40)	0.488						
Raccoon	0.02^*^ (0.00–0.12)	<0.001	0.76 (0.35–1.69)	0.506						
Environment	0.06 (0.01–0.22)	<0.001	0.48 (0.22–1.05)	0.066						

a *A random intercept was used to account for clustering of isolates obtained from the same raccoon or swine manure pit. Variance components were as follows: IncX1-1 5.12 (95%CI: 0.94–27.80); IncI1-Igamma 3.28 (95%CI: 0.15–69.86); IncFIIS 0.44 (95%CI: 0.00–224.40); ColRNAI 7.68 (95%CI: 1.91–30.80)*.

b *Adjusted for year of sampling*.

c *Exact logistic regression model*.

d *Ordinary logistic regression model*.

* *Median unbiased estimates obtained with exact logistic regression*.

*Bold values highlight global p-values of each regression model*.

**Table 6:** Contrasts from logistic regression models assessing the statistically significant associations between source type and the occurrence of select antimicrobial resistance genes and predicted plasmids in *Salmonella enterica* isolates collected from raccoons, humans, livestock, and environmental sources in southern Ontario, Canada 2011–2013 (*n* = 608).

	**IncX1-1** ^**a**^		**IncFIIS** ^**a**^		**ColRNAI** ^**a**^		**ColpVC** ^**b**^		**IncFiip96a** ^**c**^	
**Contrast**	**OR (95%CI)**	***p*-value**	**OR (95%CI)**	***p*-value**	**OR (95%CI)**	***p*-value**	**OR (95%CI)**	***p*-value**	**OR (95%CI)**	***p*-value**
Livestock vs. Raccoon	1.44 (0.55–3.77)	0.461	0.06 (0.02–0.22)	<0.001	19.07 (2.76–131.58)	0.003	23.72 (3.21–175.21)	0.002	0.01 (0.00–0.03)	<0.001
Environment vs. Raccoon	0.15 (0.03–0.71)	0.017	0.34 (0.15–0.76)	0.008	2.23 (0.36–13.87)	0.388	9.82 (1.25–77.23)	0.030	0.28 (0.14–0.52)	<0.001
Livestock vs. Environment	9.67 (2.06–45.42)	0.004	0.18 (0.08–0.42)	<0.001	8.53 (1.85–39.29)	0.006	2.41 (1.26–4.63)	0.008	0.02 (0.00–0.10)	<0.001
	***tet*(*B*)^**b**^**		***aac*(6′)−*Iaa*^**d**^**		***aph*(6)−*Id*^**b**^**		***aph*(3″)−*Ib*^**c**^**		$bla_{\text{CMY}-2}^{c}$	
	**OR (95%CI)**	***p*** **-value**	**OR (95%CI)**	***p*** **-value**	**OR (95%CI)**	***p*** **-value**	**OR (95%CI)**	***p*** **-value**	**OR (95%CI)**	***p*** **-value**
Livestock vs. Raccoon	34.03 (4.61–251.02)	0.001	1.25 (0.56–2.80)	0.587	29.73 (4.04–218.97)	0.001	36.03^*^ (6.49–∞)	<0.001	27.41^*^ (4.91–∞)	<0.001
Environment vs. Raccoon	1.64 (0.15–18.64)	0.687	2.16 (0.91–5.09)	0.079	4.76 (0.56–40.51)	0.153	2.78^*^ (0.29–∞)	0.268	2.77^*^ (0.29–∞)	0.268
Livestock vs. Environment	20.68 (4.97–85.98)	<0.001	0.58 (0.33–1.03)	0.064	6.24 (2.63–14.84)	<0.001	11.52 (3.67–58.30)	<0.001	8.77 (2.77–44.64)	<0.001

a *Multi-level model with random intercept to account for clustering of isolates obtained from the same animal or swine manure pit*.

b *Adjusted for year of sampling*.

c *Exact logistic regression model*.

d *Ordinary logistic regression model*.

* *Median unbiased estimates were obtained using exact logistic regression*.

## Discussion

A diversity of serovars and sequence types were isolated from humans, livestock, raccoons and environmental sources in our study region. Our course-grained epidemiological investigation of *Salmonella* from various sources in southern Ontario has provided insights into potential transmission between these different sources, and provides further evidence that, although raccoons have the potential to disseminate *Salmonella* and AMR to humans, their contribution appears to be minimal. Our findings build on existing work suggesting that wildlife play a largely indirect role in the transmission of *Salmonella*, serving primarily as biological intermediaries between humans, livestock and the environment, rather than acting as a primary driver or major reservoir (40–44). The identification of highly similar or identical cgMLST types (<10 allelic differences) was a rare occurrence in this population of over 600 isolates and was very specific to certain sources and serovars, highlighting potential transmission only in certain contexts (i.e., *S*. Heidelberg between poultry and humans, *S*. Newport between raccoons and soil). Likely these instances represent the acquisition of *Salmonella* by humans through undercooked poultry [for *S*. Heidelberg; (45)] or as a result of frequent exchange of *Salmonella* between raccoons and their immediate environment, as has been previously documented (25, 46). Alternatively, overlap in cgMLST types between these different sources could indicate exposure to a common source that was not identified here. Overall, we found little evidence of overlap between *Salmonella* from raccoons and human cases based on cgMLST, whereas livestock and environmental isolates from soil and water samples showed greater similarity to human isolates. The low prevalence of antimicrobial resistance among these raccoon isolates, and the rare occurrence or absence of genes conferring resistance to high-priority antimicrobials further advances the impression that raccoons are unlikely to be a primary driver or source of multi-drug resistant *Salmonella* infections for humans or livestock in this region. The selection of human isolates for inclusion in this study, however, was not random, since we included only pre-existing sequence data consisting of antimicrobial resistant human salmonellosis cases reported to public health, which merits consideration in the interpretation of our findings.

Upon closer examination of the distribution of human serovars in our study in relation to previous human *Salmonella* data from this region, there was some overlap in the top five serovars identified in our study (*S*. Enteritidis, *S*. Heidelberg, *S*. Kentucky, *S*. Typhimurium, *S*. Infantis), and the top serovars in humans previously reported by Flockhart et al. (26) in this sentinel site (*S*. Enteritidis, *S*. Typhimurium, *S*. Heidelberg, *S*. Newport, *S*. Thompson), and also at the provincial level (*S*. Enteritidis, *S*. Typhimurium, *S*. Heidelberg, *S*. Typhi, *S*. Newport; 47). The top three serovars responsible for human cases in our study, *S*. Enteritidis, *S*. Heidelberg, and *S*. Kentucky, accounted for over 65% of all human isolates in this study. The presence of *S*. Kentucky among top serovars causing human illness likely resulted from biases in the selection of human isolates for sequencing (and thus, inclusion in our study) based on phenotypic resistance. Of note, *S*. Kentucky previously accounted for <1% of human cases in an earlier study of this same FoodNet Canada sentinel site (2006–2011; 26). All human *S*. Kentucky isolates in our study were exclusively ST198, and all demonstrated phenotypic resistance to ciprofloxacin. Globally, the emergence of multi-drug resistant travel-associated *S*. Kentucky ST198 in humans is a growing concern (48); unfortunately, potential travel-related exposures among our human isolates could not be confirmed since epidemiological data concerning travel were unavailable. Apart from human isolates, the only other source containing ST198 isolates (all susceptible to ciprofloxacin) were chickens (*n* = 4/214), consistent with previous reports (48). Both *S*. Kentucky and *S*. Heidelberg were common among chicken isolates, but less common or absent in other sources (indeed, *S*. Kentucky was not isolated from raccoons). These findings are similar to previous work in this region implicating poultry as the most likely source of infection for human cases of *S*. Enteritidis and *S*. Heidelberg based on phage-typing and PFGE (26). In the present study, highly similar or identical *S*. Enteritidis ST11 and *S*. Heidelberg ST15 were identified in humans and from livestock (minimum 12 and 0 cgMLST loci, respectively). Single isolates of *S*. Enteritidis ST11 and *S*. Heidelberg ST15 identified in two different raccoons most likely represent an infection/colonization in the raccoon which originated from a poultry source, due to the apparent association with poultry isolates, along with previous work suggesting links between broiler chickens and these particular serovars in this region, and elsewhere (26, 49). With the exception of the single *S*. Heidelberg ST15 raccoon isolate that differed only at four loci (15 SNPs) from the most closely related human *S*. Heidelberg, none of the *Salmonella* isolates from raccoons clustered together with isolates from humans (based on our 10 loci threshold), thus suggesting that raccoon-human transmission of antimicrobial resistant *Salmonella* in our study populations rarely occurred, if at all.

*Salmonella* Newport was by far the most common serovar identified among raccoons, accounting for just over a third of all raccoon isolates; the two human *S*. Newport isolates were a different sequence type from those identified in raccoons, with a large number of allelic differences (>350 cgMLST loci), which suggests a different source for these human cases (possibly travel-related). Serovars that were occasionally isolated from raccoons (e.g., *S*. Typhimurium, *S*. Infantis) were commonly identified in livestock and environmental sources, suggesting widespread, and frequent dissemination of these generalist *Salmonella* serovars. Conversely, other *Salmonella* serovars were restricted to certain livestock hosts (e.g., *S*. Cerro in cattle), with a handful of water isolates that suggests sporadic environmental contamination with principally livestock-associated serovars (50, 51).

The sampling bias related to the inclusion of human isolates on the basis of demonstrated phenotypic resistance was clearly reflected in the prevalence of phenotypic multiclass resistance in the various sources (i.e., 65% of human isolates, and <15% in other sources), as well as the diversity of resistance genes present. By comparison, the prevalence of multiclass resistance among human *Salmonella* isolates identified across all of Ontario by CIPARS during the same time period was much lower, ranging between 1.5 and 37.1%, depending on the serovar of interest (52, 53). Among the remaining non-human sources, which included both phenotypically non-resistant and resistant isolates, the prevalence of multidrug resistance (3+ classes) and the diversity of resistance genes identified were highest among livestock isolates, followed by environmental isolates, and, lastly, raccoons. Although raccoons on swine farms were frequently infected with *Salmonella* [29% prevalence, as previously demonstrated by Bondo et al. (21)], antimicrobial resistance was uncommon (3.2%), and was comparable or lower than other studies of *Salmonella* in urban and suburban raccoons in Costa Rica and Japan [9–27%; (19, 22)]. Antimicrobial resistance genes were generally highest in human (e.g., *bla*_CMY−2_) and livestock sources (e.g., *tetB*) compared with raccoon and environmental isolates, consistent with a lack of direct exposure of free-ranging wildlife to antimicrobials (other than in wildlife rehabilitation settings). The predominant types of genotypic resistance identified in the subset of swine farm isolates from the previous wildlife research study were to aminoglycosides and fosfomycin (mediated by *fosA7*, but not tested phenotypically). This unusual finding of *fosA7* or *fosA7*-like genes in wildlife has recently been documented in a white-tailed eagle (*Haliaeetus albicilla*) in Poland (54), and in Andean condors (*Vultur gryphus*) in Chile (55), following its initial discovery among *S*. Heidelberg from broiler chickens in British Colombia, Canada (56). Fosfomycin, an old antibiotic that has once again regained popularity due to the emergence of multi-drug resistant (particularly ciprofloxacin-resistant) lower urinary tract infections in humans (57) is currently considered a critically important antimicrobial by the World Health Organization. Although the identification of a high prevalence (20%) of *fosA7* among raccoons in our study represents a potential public health concern, this gene was found in a similar prevalence across human, livestock and environmental samples. Fosfomycin is not widely used in veterinary medicine or animal feed, but is indicated for the treatment of infectious diseases in poultry and swine (58). The revival of fosfomycin in human and in veterinary medicine in response to the emergence of multi-drug resistance may represent a potential source of contamination of *fosA7* for wildlife and their environment, thus, continued monitoring and surveillance of this particular type of resistance is needed.

Along with the minimal overlap observed between *Salmonella* from raccoons and humans based on cgMLST typing, the absence of certain resistance genes of public health concern (e.g., *bla*_CMY−2_, *bla*_CARB−3_, *qnrS1*) in the subset of swine farm isolates from the previous wildlife study suggests that raccoons and the swine farms sampled within the Grand River watershed did not play a substantial role in the direct dissemination of high-priority antimicrobial resistant strains of *Salmonella* to humans in this region. In contrast, water isolates from this watershed, similar to livestock, contained resistance genes to numerous antimicrobial classes (e.g., *bla*_CMY−2_, *sul1, tetA, qnrS1, cmlA1*), which supports the notion that environmental exposures in this region (e.g., the use of recreational areas for swimming) may be important contributors to resistant human *Salmonella* cases, especially given the high number of conservation areas located within this watershed (*n* = 11). *Salmonella* has consistently been isolated from water sources across Canada, including those within this watershed (14, 26, 27, 59, 60); further work is needed, however, to clarify and assess the potential contribution of water sources to cases of human salmonellosis in this region.

The distribution of plasmid incompatibility groups in the dataset as a whole also varied by source for five of the six predicted plasmids assessed, providing evidence of limited transmission between different sources in this region (one would expect widespread transmission and sharing of plasmids to produce a lack of statistical association with the “source type” variable). For instance, the prevalence of colRNAI and colpVC were highest in *Salmonella* from livestock sources, whereas IncX1-1 was most prevalent in *Salmonella* from humans. In contrast, IncFiip96a was only identified in *Salmonella* from raccoon and soil isolates and these particular predicted plasmids do not appear to be associated with resistance genes, given its high prevalence (48%) along with the low prevalence of resistance in these sampling sources (3%). We did not characterize plasmid mobility, thus it is unknown whether these incompatibility groups representing plasmids have the potential to be an important factor in the circulation of antimicrobial resistance genes, genes conferring resistance to heavy metals or disinfectants, or relevant virulence factors (not assessed here).

## Limitations

As previously indicated, a major limitation of our study relates to systematic bias in the selection of human isolates for sequencing as part of public health surveillance programs. Previous work documented the presence of *S*. Thompson and *S*. Newport within the top ten serovars causing human illness in Ontario and in our study region between 2006 and 2011 (26, 47). Without a comprehensive examination of all human *Salmonella* cases in the region (including those not reported to public health authorities), it remains to be determined whether milder, non-resistant *Salmonella* infections in humans could be related to wildlife and environmental sources. For instance, *S*. Thompson and *S*. Newport were both highly prevalent serovars in raccoon and soil isolates from swine farms, both have previously been documented in water isolates in this study region (9, 26, 27), and they have also consistently been documented as top serovars causing human illness in this region (26, 47). Our collection of resistant human isolates only contained two *S*. Newport and no *S*. Thompson isolates; it remains to be seen whether other human infections with these serovars—not captured here—may be originating from wildlife and related environmental sources. In addition, associations and prevalence measures involving livestock isolates included in this study may also be impacted by selection bias, since the inclusion and continuing enrollment of farms in the FoodNet Canada surveillance program was not random, as previously discussed by Flockhart et al. (26). Similarly, the population of raccoons in our study should not be considered representative of all raccoons in the Grand River watershed, as a result of trapping animals (61), and focusing on a rural population of raccoons in agricultural areas.

Additional limitations relate to plasmid determination using incompatibility groups, and lack of characterization of plasmid mobility. Previous work has demonstrated that plasmid distribution varies by serovar (15), however, the aim of our work was to characterize patterns in the occurrence of genes and incompatibility groups representing plasmids in different sources to address potential transmission and propose primary sources. Due to the sheer number of serovars with few isolates that prevented many models from converging when serovar was included as a random intercept (data not shown), we were unable to control for the potential confounding effect of serovar on the occurrence of different plasmid types or resistance genes in different sources, which may be a particularly important factor for plasmids that are non-mobile or that have limited mobility.

Finally, our use of “livestock” as a broad category in statistical models to capture chicken, cattle, and swine isolates prevented us from identifying species-specific differences. However, this study provides a preliminary examination of potential transmission of *Salmonella* and AMR determinants between broad groups of animals, and the lumping of livestock into one category avoided potential misclassification of isolates associated with the surveillance-collected fecal samples from multi-species farms. Among these surveillance-collected isolates, fecal samples may have been obtained fresh, or from standing pooled manure piles; in the future, efforts focused on the impact of these sampling methods will be important for the utilization and interpretation of heterogeneous sampling methods and sources.

## Conclusions

This study offers new insights about the epidemiology of *Salmonella* and associated AMR from human, animal, and environmental sources in a populous region of southern Ontario. Overall, raccoons sampled on swine farms in this study were rarely infected with internationally recognized *Salmonella* lineages (39), or with antimicrobial resistant isolates (<5%). Conversely, resistance was more commonly identified in livestock and water isolates, and a number of resistance genes of public health importance (e.g., *bla*_CMY−2_, *bla*_CARB−3_, *qnrS1*) were identified in some of these sources. Although other studies have demonstrated strong links between *Salmonella* isolates from wildlife and humans (17, 62, 63), our assessment of microbial population structure based on cgMLST suggests that raccoons captured on swine farms are an unlikely source of resistant *Salmonella* for humans in this study region. However, the occurrence of certain livestock-associated *Salmonella* serovars and various resistance determinants in raccoons supports their role as sentinels and potential disseminators of these organisms within the environment. Further research, including a comprehensive examination of all human cases (including phenotypically susceptible ones), and examination of additional raccoon populations (both urban and rural) may help to provide additional insight into the potential role of raccoons in human *Salmonella* infections.

## Data Availability Statement

Previous epidemiological data from the wildlife study are available from the Agri-environmental Research Data Repository: http://dx.doi.org/10.5887/AERDR/10864/12074. Sequence data for isolates from the wildlife study have been deposited to Genbank and are available under BioProject PRJNA745182; accession numbers for those isolates are available in Supplementary Information. Interested researchers who would like to access FoodNet Canada or Public Health Ontario data will need to meet their requirements and make a data sharing agreement with those organizations.

## Ethics Statement

Ethical review and approval was not required for the study on human participants in accordance with the local legislation and institutional requirements. Written informed consent for participation was not required for this study in accordance with the national legislation and the institutional requirements. The animal study was reviewed and approved by Research Ethics Board, University of Guelph. Written informed consent was obtained from the owners for the participation of their animals in this study.

## Author Contributions

NV, BH, DP, CJ, EP, RR-S, and MM were involved in the conceptualization of the study. Funding acquisition was performed by CJ, DP, MM, EP, RR-S, and VA. DP, MM, EP, RR-S, CJ, and NR supervised the project. Curation of previously collected data was performed by KB, KZ, SK, AB, and JR. KB and SA were involved in study design of and field data collection for the previous wildlife study. NJ oversaw culture of isolates, and microbiological methodology for previous wildlife study. NV, AB, MM, JR, KZ, SK, VA, AM, EP, CJ, and JN assisted with acquisition of sequence data from public health, and coordination of sequencing of wildlife study isolates. Curation, cleaning, and analysis of WGS data was performed by NV, BH, and AV. Data analyses were performed by NV, BH, and AV, with assistance from DP and MM. Writing of the original draft was performed by NV. All authors have read and approved the final manuscript.

## Funding

Funding was provided by the Ontario Ministry of Agriculture, Food, and Rural Affairs (OMAFRA), through the Ontario Agri-Food Innovation Alliance (UofG2016-2642). NV received stipend funding from the Ontario Veterinary College, University of Guelph and a National Sciences and Engineering Research Council Postgraduate Scholarship-Doctoral.

## Conflict of Interest

The authors declare that the research was conducted in the absence of any commercial or financial relationships that could be construed as a potential conflict of interest.

## Publisher's Note

All claims expressed in this article are solely those of the authors and do not necessarily represent those of their affiliated organizations, or those of the publisher, the editors and the reviewers. Any product that may be evaluated in this article, or claim that may be made by its manufacturer, is not guaranteed or endorsed by the publisher.

## Supplementary Material

Supplementary File 1 Core-genome multi-locus sequence types, legacy multi-locus sequence types, cgMLST serovars, and gene accession numbers for *Salmonella enterica* isolates from previous wildlife study.

Supplementary Figure 1 Population structure of 608 *Salmonella enterica* isolates from raccoons, livestock, humans, and environmental sources in southern Ontario, Canada based on 3,002-loci cgMLST scheme from Enterobase, showing distribution of detailed sampling sources.

Heatmap of all serovars identified in humans and their proportional occurrence in *Salmonella enterica* isolates from raccoons, livestock, and environmental sources in southern Ontario, Canada 2011–2013 (n=608). Counts of isolates and serovars are indicated on the left. Heatmap indicates the proportion of isolates from each source type in common with human serovars, with darker shading representing a higher proportion. Except for human sources, row totals of proportions do not add up to 100% since only serovars identified in human isolates are presented here.

Distribution of *Salmonella enterica* legacy multi-locus sequence types for isolates obtained from raccoons, humans, livestock, and environmental sources in southern Ontario, Canada 2011–2013 (n=608).

Test sensitivity and specificity for in silico identification of acquired antimicrobial resistance genes in *Salmonella enterica* isolates from raccoons, humans, livestock, and environmental sources in southern Ontario, Canada 2011–2013 (n=608).
