# Supplementary figures and images for: Rural Raccoons (Procyon lotor) Not Likely to Be a Major Driver of Antimicrobial Resistant Human Salmonella Cases in Southern Ontario, Canada: A One Health Epidemiological Assessment Using Whole-Genome Sequence Data
Source: Front Vet Sci. 2022 Feb 25;9:840416. doi: 10.3389/fvets.2022.840416 (PMC8914089; doi:10.3389/fvets.2022.840416)

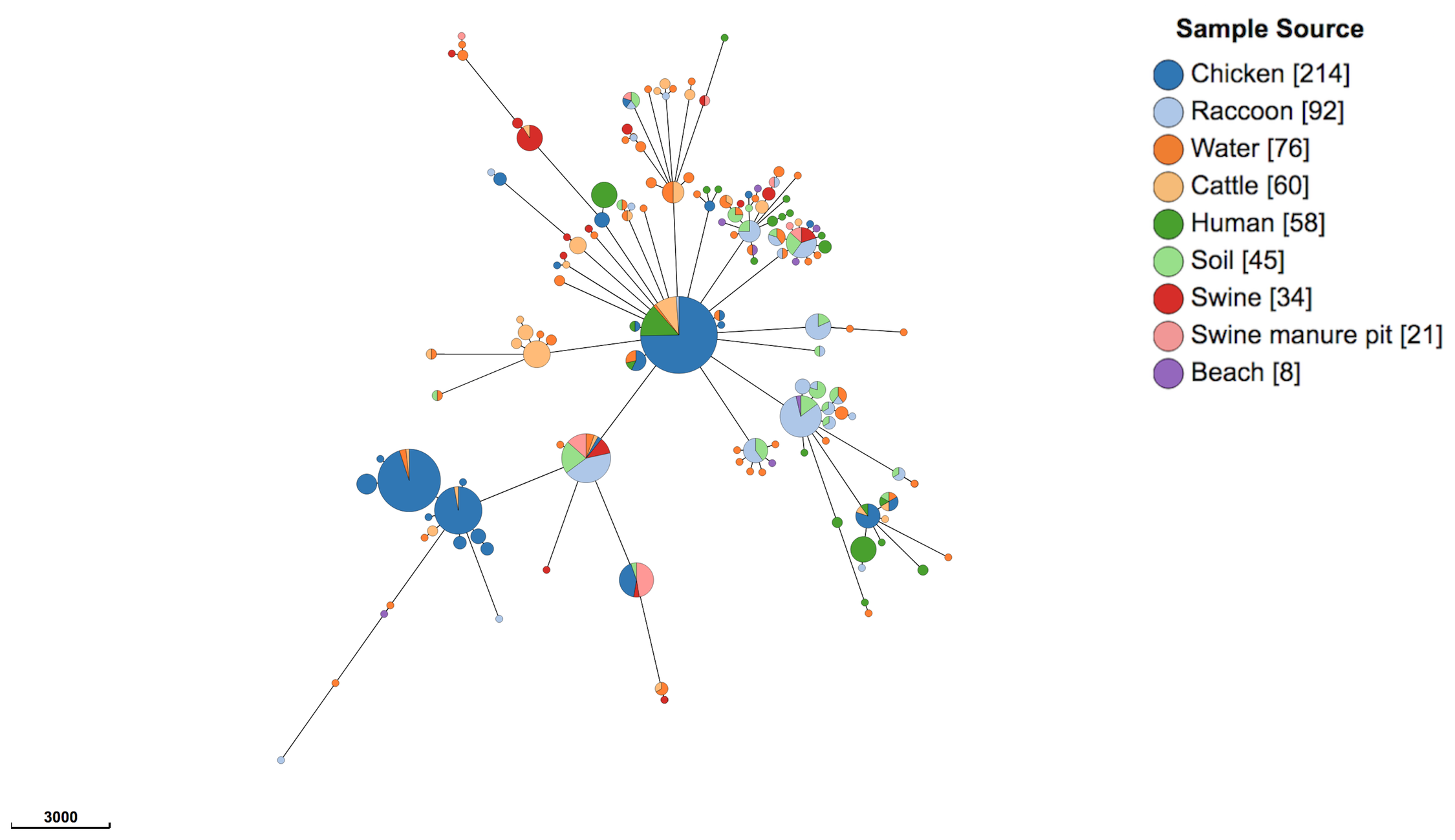

Supplement: Supplementary Figure 1 — Population structure of 608 Salmonella enterica isolates from raccoons, livestock, humans, and environmental sources in southern Ontario, Canada based on 3,002-loci cgMLST scheme from Enterobase, showing distribution of detailed sampling sources. [file Image_1.TIF]
