# Supplementary material for: Rural Raccoons (Procyon lotor) Not Likely to Be a Major Driver of Antimicrobial Resistant Human Salmonella Cases in Southern Ontario, Canada: A One Health Epidemiological Assessment Using Whole-Genome Sequence Data
Source: Front Vet Sci. 2022 Feb 25;9:840416. doi: 10.3389/fvets.2022.840416 (PMC8914089; doi:10.3389/fvets.2022.840416)
Supplement: Supplementary file 3 — Heatmap of all serovars identified in humans and their proportional occurrence in Salmonella enterica isolates from raccoons, livestock, and environmental sources in southern Ontario, Canada 2011–2013 (n=608). Counts of isolates and serovars are indicated on the left. Heatmap indicates the proportion of isolates from each source type in common with human serovars, with darker shading representing a higher proportion. Except for human sources, row totals of proportions do not add up to 100% since only serovars identified in human isolates are presented here. [file Table_1.DOCX]

**Supplementary Table 1. Heatmap of all serovars identified in humans and their proportional occurrence in *Salmonella enterica* isolates from raccoons, livestock, and environmental sources in southern Ontario, Canada 2011–2013 (n=608). Counts of isolates and serovars are indicated on the left. Heatmap indicates the proportion of isolates from each source type in common with human serovars, with darker shading representing a higher proportion. Except for human sources, row totals of proportions do not add up to 100% since only serovars identified in human isolates are presented here.**
