# Supplementary material for: Rural Raccoons (Procyon lotor) Not Likely to Be a Major Driver of Antimicrobial Resistant Human Salmonella Cases in Southern Ontario, Canada: A One Health Epidemiological Assessment Using Whole-Genome Sequence Data
Source: Front Vet Sci. 2022 Feb 25;9:840416. doi: 10.3389/fvets.2022.840416 (PMC8914089; doi:10.3389/fvets.2022.840416)
Supplement: Supplementary file 4 — Distribution of Salmonella enterica legacy multi-locus sequence types for isolates obtained from raccoons, humans, livestock, and environmental sources in southern Ontario, Canada 2011–2013 (n=608). [file Table_2.DOCX]

**Supplementary Table 2: Distribution of *Salmonella* *enterica* legacy multi-locus sequence types for isolates obtained from raccoons, humans, livestock, and environmental sources in southern Ontario, Canada 2011-2013 (n=608)**

| **Sequence type^a^ (*Serovar*)** | **Source type** | | | | | | **Total^d^ (%)** |
| --- | --- | --- | --- | --- | --- | --- | --- |
|  | **Raccoon (n=92)** | **Human (n=58)** | **Environ-mental^b^ (n=129)** | **Cattle (n=60)** | **Swine^c^ (n=55)** | **Chicken (n=214)** |  |
| ST152 (*S.* Kentucky) | 0 | 0 | 3 | 4 | 0 | 109 | 116 (19.1) |
| **ST15 (*S.*** **Heidelberg)** | 1 | 13 | 4 | 8 | 0 | 69 | 95 (15.6) |
| ST350 (*S.* Newport) | 31 | 0 | 17 | 0 | 0 | 0 | 48 (7.9) |
| ST13 (*S.* Agona) | 16 | 0 | 11 | 1 | 9 | 1 | 38 (6.3) |
| **ST19 (*S.*** **Typhimurium)** | 7 | 1 | 15 | 4 | 5 | 1 | 33 (5.4) |
| **ST32 (*S.*** **Infantis)** | 9 | 3 | 12 | 1 | 6 | 1 | 32 (5.3) |
| ST11(*S.* Enteritidis) | 1 | 14 | 2 | 3 | 0 | 10 | 30 (4.9) |
| ST367 (*S.* Cerro) | 0 | 0 | 3 | 19 | 0 | 0 | 22 (3.6) |
| ST638 (*S.* Livingstone) | 0 | 0 | 1 | 0 | 10 | 8 | 19 (3.1) |
| ST26 (*S.* Thompson) | 6 | 0 | 10 | 0 | 0 | 0 | 16 (2.6) |
| **ST198 (Kentucky)** | 0 | 11 | 0 | 0 | 0 | 4 | 15 (2.5) |
| ST592 (*S.* Worthington) | 0 | 0 | 0 | 1 | 12 | 0 | 13 (2.1) |
| ST654 (*S.* Give) | 0 | 0 | 8 | 4 | 0 | 0 | 12 (2.0) |
| ST404 (*S.* Paratyphi B var. Java) | 9 | 0 | 2 | 0 | 0 | 0 | 11 (1.8) |
| **ST96 (*S.*** **Schwarzengrund)** | 1 | 0 | 4 | 0 | 2 | 0 | 7 (1.1) |
| ST684 (*S.* Uganda) | 0 | 0 | 0 | 5 | 1 | 0 | 6 (1.0) |
| **ST10 (*S.*** **Dublin)** | 0 | 2 | 0 | 0 | 0 | 0 | 2 (0.3) |
| **ST45 (*S.*** **Newport)** | 0 | 1 | 1 | 0 | 0 | 0 | 2 (0.3) |
| **ST65 (*S.*** **Brandenburg)** | 0 | 0 | 0 | 0 | 2 | 0 | 2 (0.3) |

^a^ Sequence types (STs) determined using 7-loci Achtman scheme. Bolded STs represent internationally recognized sequence types (Monte et al., 2019).

^b^ Includes water isolates obtained through FoodNet Canada surveillance, as well as soil isolates obtained from a wildlife study (Bondo et al., 2016a).

^c^ Includes swine fecal and manure isolates collected through FoodNet Canada surveillance, as well as swine manure pit isolates obtained from a wildlife study (Bondo et al., 2016a).

^d^ Other STs identified within 5 or fewer isolates were: ST11 (*S.* Enteritidis; n=2), ST14 (*S.* Seftenburg; n=2), ST118 (*S.* Newport; n=1), ST138 (*S.* Montevideo; n=3), ST150 (*S.* Bovismorbificans; n=1), ST155(*S.* London; n=3), ST1565 (*S.* Tennessee; n=1), ST163 (*S.* Newport; n=1), ST1675 (*S.* Oranienburg; n=1), ST1838 (I 1,4,[5],12:b:-, n=1), ST2076 (*S.* Typhimurium, n=2), ST214 (*S.* Litchfield, n=2), ST22 (*S.* Braenderup; n=4), ST23 (*S.* Oranienburg; n=4), ST2507 (*S.* Holcomb; n=1), ST27 (*S.* Saintpaul; n=1), ST2848 (IIIb 11:k:z53; n=2), ST29 (*S.* Stanley; n=1), ST2937 (*S.* Infantis; n=1), ST308 (*S.* Poona; n=5), ST309 (*S.* Kiambu; n=2), ST329 (*S.* Ohio; n=3), ST33 (*S.* Hadar; n=5), ST34 (*S.* Typhimurium; n=4), ST343 (*S.* Chester; n=1), ST36 (*S.* Typhimurium; n=1), ST3790 (*S.* Heidelberg; n=4), ST40 (*S.* Derby; n=4), ST405 (*S.* Hartford; n=5), ST413 (*S.* Mbandaka; n=5), ST435 (*S.* Berta; n=1), ST469 (*S.* Rissen; n=1), ST544 (*S.* Molade; n=1), ST639 (*S.* Orion; n=2), ST64 (*S.* Anatum; 5), ST72 (*S.* Derby; n=1).

Seven isolates (raccoon-, soil-, water-, swine-, chicken-) are not included as they were not typeable.
