# Supplementary material for: Rural Raccoons (Procyon lotor) Not Likely to Be a Major Driver of Antimicrobial Resistant Human Salmonella Cases in Southern Ontario, Canada: A One Health Epidemiological Assessment Using Whole-Genome Sequence Data
Source: Front Vet Sci. 2022 Feb 25;9:840416. doi: 10.3389/fvets.2022.840416 (PMC8914089; doi:10.3389/fvets.2022.840416)
Supplement: Supplementary file 5 — Test sensitivity and specificity for in silico identification of acquired antimicrobial resistance genes in Salmonella enterica isolates from raccoons, humans, livestock, and environmental sources in southern Ontario, Canada 2011–2013 (n=608). [file Table_3.DOCX]

**Supplementary Table 3. Test sensitivity and specificity for *in silico* identification of acquired antimicrobial resistance genes in *Salmonella enterica* isolates from raccoons, humans, livestock, and environmental sources in southern Ontario, Canada 2011*–*2013 (n=608)**

| **Antimicrobial class** | **Test Sensitivity^a^ (95%CI)** | **Test Specificity^a^ (95% CI)** |
| --- | --- | --- |
| Aminoglycoside | 95.2% (89.9*–*98.2%) | 85.5% (82.0*–*88.5%) |
| Beta-lactam | 97.4% (92.7*–*99.4%) | 99.6% (98.5*–*99.9%) |
| Macrolide | 100% (0.0*–*100%)* | 100% (99.4*–*100%)* |
| Sulfonamide | 89.1% (76.4*–*96.4%) | 96.6% (94.7*–*97.9%) |
| Phenicol | 100% (83.1*–*100%)* | 99.6% (98.8*–*99.9%) |
| Tetracycline | 98.0% (94.4*–*99.6%) | 99.5% (98.4*–*99.9%) |
| Overall^b^ | 96.3% (94.2*–*97.8%) | 97.0% (96.4*–*97.6%) |

^a^ Phenotypic antimicrobial resistance test results were considered the gold standard. Detection of 15 antimicrobials performed using the CMV3AGNF panel from National Antimicrobial Resistance Monitoring System (NARMS; Sensititre, Thermo Scientific). *In silico* acquired resistance genes detected using CARD-Resistance Gene Identifier.

^b^ Raw counts for all isolates and antimicrobials were pooled together.

*One-sided 97.5% confidence interval.
